# Implant-Based Breast Reconstruction after Risk-Reducing Mastectomy in BRCA Mutation Carriers: A Single-Center Retrospective Study

**DOI:** 10.3390/healthcare11121741

**Published:** 2023-06-13

**Authors:** Emanuele Cammarata, Francesca Toia, Matteo Rossi, Calogero Cipolla, Salvatore Vieni, Antonino Speciale, Adriana Cordova

**Affiliations:** 1 Plastic and Reconstructive Surgery Unit, Department of Surgical, Oncological and Oral Sciences (Di.Chir.On.S.), University of Palermo, Via del Vespro 129, 90127 Palermo, Italy; 2 Oncological Surgery Unit, Department of Surgical, Oncological and Oral Sciences (Di.Chir.On.S.), University of Palermo, Via del Vespro 129, 90127 Palermo, Italy

**Keywords:** breast cancer, BRCA mutation, risk-reducing mastectomy, breast reconstruction, direct-to-implant breast reconstruction, prepectoral breast reconstruction, acellular dermal matrix

## Abstract

Women with BRCA gene mutations have a higher lifetime risk of developing breast cancer. Furthermore, cancer is usually diagnosed at a younger age compared to the wild-type counterpart. Strategies for risk management include intensive surveillance or risk-reducing mastectomy. The latter provides a significant reduction of the risk of developing breast cancer, simultaneously ensuring a natural breast appearance due to the preservation of the skin envelope and the nipple-areola complex. Implant-based breast reconstruction is the most common technique after risk-reducing surgery and can be achieved with either a submuscular or a prepectoral approach, in one or multiple stages. This study analyzes the outcomes of the different reconstructive techniques through a retrospective review on 46 breasts of a consecutive, single-center case series. Data analysis was carried out with EpiInfo version 7.2. Results of this study show no significant differences in postoperative complications between two-stage tissue expander/implant reconstruction and direct-to-implant (DTI) reconstruction, with DTI having superior aesthetic outcomes, especially in the prepectoral subgroup. In our experience, the DTI prepectoral approach has proven to be a safe and less time-consuming alternative to the submuscular two-stage technique, providing a pleasant reconstructed breast and overcoming the drawbacks of subpectoral implant placement.

## 1. Introduction

Patients with mutations in BRCA 1 and BRCA 2 genes have an increased chance of developing breast cancer, with a reported lifetime risk ranging from 56% to 84%. The diagnosis is often made at a younger age if compared to the healthy non-carrier population [1,2,3,4,5,6,7]. BRCA-mutated patients can undergo a close clinical and instrumental follow-up, aiming at early diagnosis, or can opt for risk-reducing mastectomy (RRM), with the latter becoming progressively popular after 2013 due to the so-called “Angelina Jolie effect” [8]. The comparison between the two options has shown that risk-reducing surgery, eliminating the potential source of the disease, provides a better protection against breast cancer than intensive screening alone [9,10,11], decreasing the risk by 90–95% [12,13,14]. In the setting of risk-reducing surgery, nipple-sparing mastectomy (NSM) is considered the technique of choice, especially for patients with mild-to-moderate breast size, thanks to its superior cosmetic results and improved patient-reported satisfaction [15,16]. Skin-reducing mastectomy (SRM) is a feasible option in case of large breasts that require correction of ptosis [17]. Almost every patient who undergo risk-reducing mastectomy ask for breast reconstruction, with the majority of them opting for implant-based approaches instead of flap-based or combined ones [18,19,20,21]. Implants can be placed either over or under the pectoralis major muscle, in two stages or in a single-stage procedure, also referred as direct-to-implant (DTI) reconstruction [22]. However, the ideal timing of breast reconstruction and the optimal location of prosthetic implants are still debated and change over time following the advancements in reconstructive surgery [23]. Techniques have gradually shifted from two-stage fully submuscular tissue expander (TE)-assisted reconstruction to single-stage partially submuscular (dual-plane) and prepectoral reconstruction. Therefore, in recent years, the direct-to-implant approach has become widespread, representing a true paradigm shift in breast reconstruction [24]. This is mainly due to several factors: the improvement in mastectomy techniques and surgical skills, the growing trend in skin- and nipple-sparing mastectomies [25], the introduction of hybrid breast reconstruction with the use of additional ancillary procedures like fat grafting [26] and the development of new tissue perfusion assessment tools such as indocyanine green (ICG) fluorescence angiography [27]. Moreover, the advent of bioengineered acellular dermal matrices (ADM) has represented another critical aspect that contributed to this shift [28,29,30]. ADMs are ready-to-use, non-immunogenic biocompatible materials that integrate with the host’s tissues and promote tissue vascularization and cellular proliferation. The introduction of ADMs in the surgeon’s armamentarium have allowed a dramatic increase in the reconstructive potentialities in the field of breast reconstruction, thanks to their ability to provide additional coverage to the implants, especially if they are placed directly under the subcutaneous tissue, to actively shape the lower pole and to reduce the rates of capsular contracture [31].

To date, the choice of the appropriate procedure among the broad reconstructive scenario (one-stage vs. two-stage, prepectoral vs. partially or totally subpectoral) is made upon surgeon’s preferences, but must be based on patient’s requirements, careful patient selection and meticulous evaluation of potential risk factors [32]. 

The aim of this study is to analyze, through a retrospective analysis of a consecutive single-center case series, the comparative outcomes of the different implant-based reconstructive techniques, and orient clinical decisions in the setting of breast reconstruction after risk-reducing mastectomy.

## 2. Materials and Methods

### 2.1. Study Design and Patient Selection

This study was designed as a single-center case series and was performed through a retrospective review of 32 consecutive BRCA-mutated women (46 breasts) who underwent bilateral or unilateral risk-reducing mastectomy and subsequent implant-based breast reconstruction at the authors’ institution (Plastic and Reconstructive Surgery Unit, University Hospital “Paolo Giaccone”, Palermo) from January 2018 to March 2022. The study received the approval of the Ethical Committee of the University Hospital “Paolo Giaccone” of Palermo. 

### 2.2. Data Collection

Demographic, clinical, intraoperative and postoperative characteristics of patients were collected through a retrospective screening of medical records (see Table 1, Table 2 and Table 3). Major complications were defined as complications that resulted in implant loss and/or could not be managed conservatively, requiring additional surgical procedures under general anesthesia. Pain intensity was recorded daily from the first postoperative day through a self-reported pain assessment scale, the Numerical Rating Scale (NRS), whose validity is supported in the literature [33,34]. Average postoperative pain was defined as the mean of patient-reported pain scores in the first three postoperative days. Patient-reported satisfaction was evaluated 6 months postoperatively, when patients were asked to answer to the question “How much are you satisfied with the overall result of your breast reconstruction?”, giving a score ranging from 1 (minimally satisfied) to 10 (fully satisfied). Similarly, surgeon-reported satisfaction was assessed through a survey administered to ten experienced plastic surgeons who did not participate to the operation, where they were asked to evaluate the cosmetic result of the reconstruction in a rating scale ranging from 0 (worst result) to 10 (best result). In this study, the surgeon-reported outcome is defined for each patient as the mean of surgeon-reported satisfaction scores.

### 2.3. Statistical Analysis

Statistical analysis was carried out with EpiInfo software version 7.2.4.0 (Epi Info™, CDC, Division of Health Informatics & Surveillance, Center for Surveillance, Epidemiology & Laboratory Services, 2020). In descriptive statistics, mean, standard deviation and range were reported for continuous variables, whereas frequency and percentage were listed for categorical variables. The Welch–Satterthwaite T-test was used to analyze means of continuous variables and a two-tailed Fisher’s exact test was used to compare frequencies of categorical variables. Contingency tables and odds ratios (OR) were used to measure the association between risk factors and the outcome of interest. Concordance between quantitative variables was calculated with Pearson’s correlation coefficient (R). Statistical significance was set at *p* < 0.05.

### 2.4. Surgical Indications

- In mild-to-moderate size breasts with no ptosis, risk-reducing mastectomy was preferentially carried out through a conventional NSM with an inframammary fold (IMF) approach.
- In cases of medium-sized breasts with additional ptosis, an inferior hemi-periareolar incision was chosen. A superiorly based nipple-areola complex (NAC) adipodermal flap was raised and a circumferential region around the NAC was dehepitelialized with the purpose of performing a concomitant periareolar pexis.
- In large and ptotic breasts, risk-reducing mastectomy was performed through an SRM, in order to provide a simultaneous mastopexy in addition to the preservation of the NAC. A bipedicled superiorly and-inferiorly based NAC adipodermal flap was raised to provide additional coverage to the underlying implant.
- We never performed primary free NAC grafting in our series, because we always relied, even in larger breasts, on the improved vascular supply provided by the bipedicled NAC-bearing flap.

Risk-reducing mastectomy was performed following the anatomical plane of the superficial fascia dividing the subcutaneous tissue from the underlying breast parenchyma, in order to remove as much gland as possible [35,36]. Sharp dissection with cold scissors or blade was preferred over monopolar electrocautery in order to avoid potential heat-induced injury to mastectomy flaps. Intraoperatively, perfusion of NAC and mastectomy flaps was evaluated clinically through direct assessment of skin quality (color, amount of preserved subcutaneous fat, lack of dermal layer exposure), temperature, bleeding of incision edges and capillary refill [37,38]. If perfusion was uncertain, skin viability was confirmed with an infracyanine green-photodynamic eye (IFCG-PDE) imaging system (PDE, Hamamatsu Photonics K.K., Hamamatsu, Japan) [39,40]. IFCG is a solution that contains the same fluorophore found in indocyanine green (ICG) but differs because is iodine-free and iso-osmolar with blood. We preferred IFCG because it has the same properties as ICG and can also be safely employed in patients allergic to iodine, showing a more favorable toxicity profile [41]. Mastectomy flap thickness was evaluated as well. In case of inadequate thickness of the residual mastectomy flaps (<10 mm), poor skin perfusion regardless of the thickness, significant tension in wound closure or other conditions that could potentially jeopardize tissue vascularization, DTI reconstruction was abandoned in favor of a two-stage procedure.

Then, the reconstruction proceeded as follows:

- In cases of two-stage submuscular reconstruction, a tissue expander (TE) was placed in a pocket dissected under the pectoralis major muscle. Expansion was carried out every week during the postoperative course. When the desired volume of the submuscular pocket was reached, the TE was removed and replaced with a permanent implant during a secondary surgery.
- In one-stage dual-plane reconstruction, a partially submuscular pocket was created. The implant was placed under the pectoralis major muscle and covered in its superior two thirds by the muscle and in its inferior third by a bovine/porcine ADM sling (SurgiMend^®^ PRS, Integra LifeSciences, Plainsboro, New Jersey or Native^®^, Decomed S.r.l., Venezia, Italy) sutured superiorly to the inferior margin of the muscle and inferiorly to the rectus sheath. This provided coverage of the lower pole of the implant. Alternatively, Bostwick’s autoderm technique was employed for the same purpose [42]. The choice between the two options was made upon surgeon’s preferences and availability of viable dermal flaps.
- If one-stage ADM-assisted prepectoral reconstruction was performed, a pre-shaped porcine ADM sheet (Braxon^®^, Decomed S.r.l., Venezia, Italy) was rehydrated in sterile saline for about 5 to 10 min. Then, it was put on a sterile desk and wrapped around the implant. The edges of the matrix were sutured, and the excess parts were trimmed. The enveloped implant was placed above the pectoralis major muscle and anchored to the chest wall through 3 to 5 cardinal sutures. Additional quilting sutures were put between the ADM and the subcutaneous layer. Fixation of the ADM avoided any migration or rotation of the implant and ensured adequate contact between the collagen membrane and the surrounding vascularized tissues.
- In cases of ADM-free prepectoral reconstruction, the prosthesis was laid down on the pectoralis major muscle without further coverage.

The choice between round and anatomical implants mainly depended on breast characteristics before mastectomy and patient’s desires, preferably opting for anatomical implants if a superior lower pole projection and a more “natural” appearance was advocated.

### 2.5. Perioperative Care

Patients were asked to wear a post-surgical compression bra from the first postoperative day and for at least one month after surgery. Drains were removed when their content was lower than 30 mL per day for two consecutive days. If no complications occurred, patients were usually discharged in 5 to 7 days. Patients were followed-up at 1, 2 and 4 weeks and at 3 and 6 months postoperatively in order to detect even tardive complications and evaluate long-term clinical outcomes. 

### 2.6. Secondary Procedures

In case of unsatisfactory cosmetic results, when requested by the patient, one or more fat grafting sessions were performed in order to correct aesthetic imperfections and camouflage implant edges visible through thin mastectomy flaps. The donor areas (abdomen, flanks or thighs) were infiltrated with tumescent (Klein’s) solution and the fat was then suctioned by hand through the use of 1 to 3 mm liposuction cannulas. Then, the collected fat was processed by centrifugation for 3 min at 3000 RPM as described by Coleman [43]. Finally, oil and blood were discarded, and the purified fat was injected into the breast in a subcutaneous plane with a blunt infiltration cannula.

## 3. Results

The mean age was 49.7 ± 6.1 years (range 35–61 years). About nineteen percent of patients were obese (BMI > 30 kg/m^2^). Eighteen patients had a past history of breast cancer, fourteen received radiation therapy and ten underwent prophylactic bilateral salpingo-oophorectomy. Demographic and clinical characteristics of patients are summarized in Table 1. Regarding intraoperative characteristics, fourteen patients underwent bilateral risk-reducing mastectomy, whereas eighteen underwent unilateral risk-reducing mastectomy with contralateral therapeutic mastectomy for breast cancer, with a total amount of forty-six breasts operated on with risk-reducing intent. In the majority of cases (65.2%) the type of risk-reducing mastectomy was an NSM. Eighteen patients underwent immediate reconstruction, and fourteen patients underwent staged reconstruction with tissue expanders. The mean volume of tissue expanders was 438.9 cc (range 300–600). As concerns breast implants, the mean volume was 436.6 cc (range 240–525). Twenty implants were round and 24 were anatomical. Acellular dermal matrices were used in twenty-six reconstructions. The most used ADM was Braxon^®^ (16/26). The drainage was removed after an average period of 8.8 days (range 4–20). The length of hospitalization ranged between 4 and 24 days (median = 8 days). The majority of patients had an uneventful recovery. Six breasts received additional lipofilling, with a mean amount of injected fat of 130 cc (Table 2 and Table 3). Detailed surgical information about the forty-six operated breasts are provided in Table 4.

**Figure 1:**
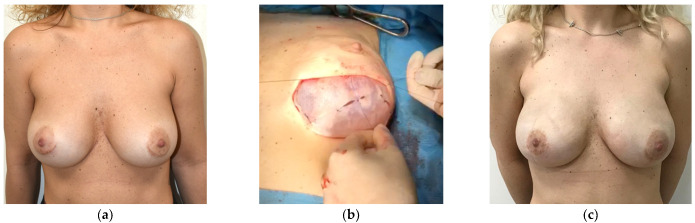
Thirty-five year-old woman with mutation in BRCA 1 gene who underwent bilateral risk-reducing nipple-sparing mastectomy with inframammary fold approach and subsequent prepectoral direct-to-implant reconstruction with ADM-wrapped implants. (**a**) Preoperative view; (**b**) intraoperative view of the left breast showing the acellular dermal matrix wrapped around the implant and anchored to the pectoralis major fascia; (**c**) postoperative view at 6 months.

**Figure 2:**
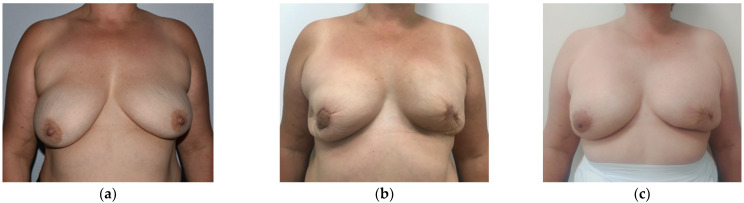
Forty-nine year-old patient with mutation in BRCA 2 gene and current diagnosis of left invasive breast cancer who underwent therapeutic skin-sparing mastectomy, contralateral risk-reducing nipple-sparing mastectomy with periareolar incision and staged submuscular tissue expanders/implants reconstruction. (**a**) Preoperative view; (**b**) postoperative view 3 months after bilateral tissue expander placement; (**c**) final result 6 months after exchange of tissue expanders with definitive implants.

Comparison between single-stage and two-stage reconstruction showed that patients with prior diagnosis of breast cancer who underwent breast and lymph node surgery or with a history of previous radiation therapy were preferentially treated with TE-assisted reconstruction at our institution (Table 5). Postoperative complications occurred in 10 breasts (5 major and 2 minor, overall complication rate = 21.7%) and were more common in the two-stage subgroup (33.3% vs. 14.3%), but this difference was not statistically significant; complications included two capsular contractures leading to implant explantation, six major skin necroses that required a return to the theatre and two minor nipple-areola-complex necroses that were managed conservatively. No seroma, hematoma or infection occurred (Table 6). In univariate analysis, none of the examined characteristic was predictive for postoperative complications at a significance level of *p* < 0.05. Nevertheless, although not significant, staged reconstruction (OR = 2.85), active smoking (OR = 9.66), previous hormonal therapy (OR = 4.84), radiotherapy (OR = 2.27), axillary surgery (sentinel lymph node biopsy and axillary lymph node dissection) (ORs = 4.81 and 3.91) or bilateral salpingo-oophorectomy (OR = 2.85) and the presence of occult cancer in risk-reducing mastectomy specimens (OR = 3.91) were associated with the highest chance of developing complications during the postoperative course (Table 7). Pearson’s Correlation coefficient (R) showed a strong positive correlation between patient-reported satisfaction (mean = 7.3) and surgeon-reported outcome (mean = 6.5) assessed at the 6-month follow-up (R = 0.9166, *p* = 0.001361). Some clinical cases are shown in Figure 1 and Figure 2.

## 4. Discussion

Our study shows that, although non suitable for all cases, DTI prepectoral breast reconstruction can be considered a safe and convenient alternative to staged breast reconstruction if performed in carefully selected patients, with a meticulous surgical technique and an accurate intraoperative evaluation of skin flaps perfusion. It is minimally invasive, provides a natural-appearing breast with higher patient-reported satisfaction and no significant increase in terms of postoperative complications, simultaneously avoiding the additional costs and visits related to tissue expander placement.

In the field of risk-reducing surgeries, there is no “standard” operation and a huge variety of techniques have been developed, differing for NAC and/or skin preservation and type of incision. The most common techniques are NSM and SRM [44]. In this series, NSM was the most common type of risk-reducing mastectomy, accounting for about two-thirds of patients, and we preferentially adopted an inframammary fold approach. In large and ptotic breasts (30.4%), we preferred a SRM through a wise pattern incision and a bipedicled adipodermal NAC flap. In fact, in these patients, NSM is usually contraindicated due to the significant risk of NAC necrosis [45]. Conversely, our technique proved to be safe in terms of NAC survival: we reported no cases of NAC necrosis after SRM. We experienced four cases of major skin necrosis (8.7%) in patients who underwent SRM and subsequent ADM-assisted PBR, but none of them ended in reconstructive failure thanks to the protection ensured by the bipedicled adipodermal flap, which provided complete vascularized coverage of the implant, preventing its direct exposure. Caputo et al. and Maruccia et al. described similarly low rates of skin necrosis (6.1% and 8.7%, respectively) after SRM and no cases of implant loss, thanks to the combination of an ADM with an inferior dermal flap in PBR [46,47]. 

In our series, when feasible, an immediate breast reconstruction (IBR) was preferred over a staged one. The literature suggests that IBR provides many advantages: a reduced operating time, a shorter length of hospitalization [48,49,50] and the avoidance of the multiple visits needed for tissue expansion [51,52,53,54,55]. Additionally, an immediate reconstruction increases women’s quality of life after risk-reducing surgery, favorably impacts on patient-reported satisfaction and psychological well-being [56,57,58,59] and is superior in terms of cosmetic results [60]. Finally, in case of PBR, the result is a more “natural” breast (the implant is placed in the same anatomical compartment formerly occupied by the surgically excised parenchyma) [25,37,51,61,62,63,64], with better lower pole projection [65,66,67], enhanced definition of IMF [68,69,70,71] and a more accurate predictability in size and symmetry [72].

In this study, patients who underwent IBR had a shorter hospital stay (mean 8.6 vs. 10.4 days), a shorter drain duration (mean 8.4 vs. 9.4 days), a lower drain amount and less postoperative pain (3.06 vs. 3.67) in comparison to those who were treated with a two-stage breast reconstruction. Both surgeon-reported outcome (7.2 vs. 5.8) and patient-reported satisfaction (7.5 vs. 7.0) were superior in the single-stage group. Our findings confirm those reported in the literature: in two different studies, Casella et al. and Cattelani et al. showed higher Q scores in patients who had a prepectoral IBR in comparison with patients treated with dual-plane IBR or staged TE-assisted reconstruction [73,74]. Bernini et al. reported that the surgeon’s judgment on aesthetic outcome was excellent in 91% of prepectoral reconstructions and 65% of subpectoral ones and this finding was coherent with patient subjective perception [25].

However, in our cohort, a strict adherence to rigorous inclusion criteria was paramount before proceeding to an IBR, especially if a prepectoral approach was chosen, in order to prevent complications. In fact, due to the partially or totally subcutaneous positioning of the implant, any skin-related problem could lead to implant exposure and therefore compromise the reconstructive outcome [75]. In our experience, the ideal candidates were patients with small-to-moderate and non-ptotic breasts [74,76,77,78,79], no associated comorbidities and an adequate thickness of mastectomy flaps. Regarding obesity, a high BMI is generally considered a relative contraindication to IBR. It lowers the chance of flap viability and increases the overall complication rate [19,24,37,77,80,81,82], particularly the probability of seroma occurrence [19,83,84]. In our retrospective study the mean BMI in patients who underwent IBR was 24.6 kg/m^2^. Differently from the literature, we also chose to include for IBR overweight or slightly obese patients (11.1%); in this group, the major complication rate was higher (30% vs. 7.7%) but overall acceptable; of note, complications never led to implant removal or complete reconstructive failure. Similarly to us, Downs et al. widened the reconstructive indication for IBR to patients with a BMI ranging from 25 to 35 kg/m^2^, even considering mild obesity an advantage for PM, since fat patients tend to have a thicker subcutaneous layer and could benefit from better perfused skin flaps [19]. 

In our study, the choice of an IBR was also conditioned by intraoperative clinical assessment of residual skin flap thickness. In the literature, there is no unanimous consensus on the best cutoff thickness: according to Nahabedian et al., a thickness greater than 10 mm is essential to proceed to immediate PBR [77,85]. In a MRI study performed by Frey et al. on 379 NSMs, an absolute mastectomy flap thickness lower than 8 mm or a low postoperative-to-preoperative thickness ratio was strongly predictive of ischemic complications, regardless of the type of reconstruction [86]. In two other studies, it was demonstrated that a mastectomy flap thickness less than 5 and 8 mm, respectively, represents a significant risk factor for ischemic complications [87,88].

In our series, we preferred to be more conservative and conventionally adopted a cutoff of 1 cm to establish whether patients were eligible for prepectoral IBR.

Concerning tissue perfusion, in patients with uncertain flap viability, we performed instrumental assessment of skin vascularization through IFCG fluorescence and a near-infrared camera (PDE), which is considered the best tool to predict mastectomy flap survival [85], and oriented the clinical decision towards a single or two-stage reconstruction. However, despite the use of IFGG angiography, we were unable to foresee four out of the six cases of major skin necrosis. These four cases occurred bilaterally in two patients who, although they were active smokers, expressed a strong intention to be treated in a single stage with a prepectoral implant and accepted the increased risk for complications. This occurrence further highlighted the relevance of appropriate patient selection to prevent potentially harmful complications. 

In our series, two-stage TE-assisted reconstruction was restricted to patients who did not meet the inclusion criteria or in whom the mastectomy flaps were too thin or poorly perfused after ICG-PDE instrumental evaluation. In particular, active smokers, immunosuppressed patients and women with poorly controlled diabetes mellitus (HbA1c > 7.5%) were preferentially excluded from IBR and better served with a staged submuscular reconstruction [23,64,77,89]. In fact, providing as it does an additional layer between the skin and the prosthetic device, a two-stage subpectoral reconstruction was a safer and most appropriated alternative in patients at high risk of postoperative complications and implant exposure [90,91,92]. However, this reconstruction has many drawbacks related to the detachment and the manipulation of the pectoralis major, such as animation deformity [19,66,93,94,95,96], upper implant displacement [37], persistent postoperative pain [97] and loss of muscle function with shoulder impairment [51,64,79,94,98,99]. Moreover, it allows for a suboptimal aesthetic outcome, with a final result that consists of a flat and “contrived” breast mound with low projection and no natural ptosis, due to the constriction exerted by the muscle on the underlying implant [62,100]. A potential advantage of TE-assisted reconstruction is that lipofilling can be performed at the same time as inserting the definitive implant. However, in our opinion this type of reconstruction should be strictly limited to the cases mentioned above.

An important aspect to take into account is the use of ADMs in IBR. 

An ADM is a biological graft derived from human, porcine or bovine tissues that acts like a scaffold that is gradually vascularized and populated by the host’s cells. Thanks to the absence of cellular and antigenic components, an ADM is a non-immunogenic material that helps to avoid the drawbacks related to the host’s immunological response. Moreover, it overcomes the disadvantages related to autologous tissue grafts and synthetic materials, represented by a secondary donor-site morbidity and a high risk of infection, respectively [31,101].

The use of ADM has become widespread in several fields of reconstructive surgery, including burns, breast and abdominal wall reconstructive surgery and gynecologic and genitourinary surgery. Additionally, their usefulness has been demonstrated in the treatment of hidradenitis suppurativa [102]. ADMs can be used alone or, alternatively, co-grafted with split thickness skin grafts (STSGs) [103]. In their study, Lee et al. showed that the combined use has a synergistic effect and results in a better scar quality than STSG alone [104].

In breast reconstructive surgery, these matrices provide an additional envelope around the implant, creating a biological interface between the prosthesis and the surrounding tissues and preventing its direct exposure in case of wound dehiscence [94]. 

In our study, complete or partial ADM coverage was used in 22/28 immediate reconstructions. Braxon^®^ is manufactured as a pre-shaped ADM and was employed in 16 breasts for complete implant coverage in cases of immediate prepectoral breast reconstruction, while Surgimend^®^ and Native^®^ ADMs are provided as sheets and were used in 6 breasts for the coverage of the lower pole of the prosthesis in case of immediate dual-plane breast reconstruction.

In four breasts, a dual-plane reconstruction was achieved with the use of Bostwick’s autoderm technique [42]. Only two patients underwent PBR using polyurethane-coated implants without ADM support, having an uneventful postoperative course and no reported complications. This ADM-free technique simultaneously eliminated the disadvantages connected to the submuscular implant placement and the added costs related to a staged procedure and to the employment of a biological matrix [22]. Thus, although less common and more dangerous in case of skin breakdown, this procedure seems promising, and our intent is to extend its application in our future studies.

Recently, several studies highlighted the problem of increased rates of capsular contracture (CC) (as high as 20%) [72] in IBR without soft-tissue support [105,106,107,108]. However, it has been demonstrated that the adjunctive use of acellular dermal matrices has a protective role against CC [109,110,111,112,113,114,115,116,117,118], dramatically reducing its occurrence thanks to the decreased inflammatory response to the implant and the absence of direct mechanical stress over the prosthesis [119,120,121,122,123]. In a systematic review of complications following PBR, Wagner et al. reported an overall incidence of CC of 8.8%, that was further stratified into ADM-assisted (2.3%) and non-ADM assisted (12.4%) cohorts, highlighting a huge difference between the two groups [124]. In our IBR cohort, we experienced no cases of capsular contracture in the DTI group at a 6-month follow-up, both in the ADM-assisted and in the non-ADM-assisted subgroups, but this result could be related to the short follow-up. 

The overall complication rate was 21.7%. We experienced no cases of seroma, hematoma or infection. Approximately 13% of patients had a major skin or NAC necrosis. Our finding are not dissimilar to that reported by Chun et al., who described major flap necrosis in 11.8% of patients [125]. Interestingly, all patients who developed mastectomy skin necroses underwent ADM-assisted reconstruction. This is a well-known issue and is coherent with the increased rate of flap necrosis reported in the literature in ADM cohorts [126]. We had no cases of early implant explantation and only two cases (4.4%) of late implant explantation that occurred in two patients who developed capsular contracture after a two-stage submuscular reconstruction; however, this rate is significantly lower than that reported in the literature (17%) [83,127]. The reason of this low incidence in our study is that most complications were managed conservatively and did not require the implant’s removal, thanks to the additional protection provided by the ADM and/or the bipedicled adipodermal flap in immediate prepectoral reconstructions, and by the pectoralis muscle together with the ADM or the Bostwick’s autoderm in the immediate dual-plane reconstructions.

Six patients required further fat grafting as a correcting procedure. In these patients the aesthetic result was unpleasant due to poor flap quality after mastectomy and supervened postoperative skin necrosis that was managed through additional surgical operations or secondary wound healing. The defects were corrected with lipofilling, which allowed us to increase the breast volume and to camouflage the cosmetic imperfections [25,28,99,128,129].

Complications were more common in patients who underwent two-stage reconstruction (33.3% vs. 14.3%), but this difference was not statistically significant. Several other comparative studies have shown that the chance of postoperative complication in IBR does not differ significantly to the other implant-based reconstructive procedures [110,130,131,132,133,134,135,136,137,138,139,140]. In our PBR cohort, the major complication rate was comparable to that found in the remaining patients (18.1% vs. 16.7%). Notably, in the literature, PBR is generally recommended for women requiring implants <400 cc [76], while our experience deals primarily with reconstructions achieved through the use of larger devices (mean = 468.1 cc). The complication rate was slightly higher than we expected in these patients, which can be justified by the higher tension in mastectomy flaps and their reduced perfusion, produced by a mismatch between implant volume and pocket size [126,141]. Published research is conflicting regarding the occurrence of complications in this particular subset of women. Salibian et al. and Chatterjee et al. performed two systematic reviews on PBR and found similar pooled complication rates [134,142]. Conversely, other authors found an increased risk of postoperative complications such as infection, flap necrosis and implant loss [19,51,79], and a higher rate of secondary revisions, up to 87% [28,29,53,82,118,143,144]. 

Given the small sample size, no association was found between the examined risk factors and the occurrence of postoperative complications. However, although these results did not reach statistical significance, active smoking (OR = 9.66), previous hormonal therapy (OR = 4.84), prior axillary surgery and the presence of incidental breast carcinoma in the mastectomy specimen (OR = 3.91) showed the highest odds ratios and may be associated with an increased likelihood of developing complications in larger cohorts. BMI was slightly higher in patients who had complications (25.66 vs. 24.43, *p* = 0.5403), but, in a difference from the literature [139], obesity was not found to increase the probability of developing any kind of sequelae in the postoperative course (*p* = 1.000). 

Limitations of this study comprehend its retrospective nature and the relatively small number of examined patients. Further prospective studies with a larger sample size and a longer follow-up are advisable in order to overcome biases and obtain statistical results with stronger evidence. 

## 5. Conclusions

Risk-reducing mastectomy is often performed in genetically predisposed, highly demanding patients who ask for a seamless and non-mutilating reconstruction, no pectoralis major disruption with preservation of muscular strength and no need for postoperative physiotherapy with a fast return to daily activities. For these reasons, it is important to perform a minimally invasive procedure with a low complication rate and good functional and cosmetic results. Direct-to-implant PBR seems to adequately fit these requirements, representing the latest frontier in breast reconstruction and emerging as a viable alternative to TE-assisted procedures.

## Figures and Tables

**Table 1:** Demographic and clinical characteristics of the study population.

Variable	*n* = 32
**Age (mean ± SD, range) (years)**	49.7 ± 6.1 (35–61)
**BRCA mutation type (No, %)**	
Type 1	28 (87.50%)
Type 2	4 (12.50%)
**BMI (mean ± SD, range) (kg/m^2^)**	25.0 ± 4.4 (19.3–32.7)
**Obesity (BMI ≥ 30) (No, %)**	
Yes	6 (18.75%)
No	26 (81.25%)
**Smoking status (No, %)**	
Smoker	4 (12.5%)
Non-smoker	28 (87.5%)
**Alcohol consumption (No, %)**	
Yes	4 (12.5%)
No	28 (87.5%)
**Coffee consumption (No, %)**	
Yes	20 (62.5%)
No	12 (37.5%)
**Diabetes mellitus (No, %)**	
Yes	0 (0.00%)
No	32 (100.00%)
**Previous adjuvant/neoadjuvant chemotherapy (No, %)**	
Yes	10 (31.25%)
No	22 (68.75%)
**Previous radiotherapy (No, %)**	
Yes	14 (43.75%)
No	18 (56.25%)
**Previous hormonal therapy (No, %)**	
Yes	6 (18.75%)
No	26 (81.25%)
**Previous breast cancer (No, %)**	
Unilateral	14 (43.75%)
Bilateral	4 (12.50%)
None	14 (43.75%)
**Previous breast surgery (lumpectomy/quadrantectomy) (No, %)**	
Unilateral	14 (43.75%)
Bilateral	4 (12.50%)
None	14 (43.75%)
**Previous SLNB ^1^ (No, %)**	
Unilateral	8 (25.00%)
Bilateral	2 (6.25%)
None	22 (68.75%)
**Previous ALND ^2^ (No, %)**	
Unilateral	2 (6.25%)
Bilateral	0 (0.00%)
None	30 (93.75%)
**Previous ovarian cancer (No, %)**	
Yes	2 (6.25%)
No	30 (93.75%)
**Previous prophylactic BSO ^3^ (No, %)**	
Yes	10 (31.25%)
No	22 (68.75%)
**Current diagnosis of breast cancer in contralateral breast (No, %)**	
Yes	18 (56.25%)
No	14 (43.75%)

^1^ Sentinel lymph node biopsy ^2^ Axillary lymph node dissection ^3^ Bilateral salpingo-oophorectomy.

**Table 2:** Intraoperative and postoperative characteristics of the twenty-three operated breasts.

Variable	*n* = 46
**Type of risk-reducing mastectomy (No, %)**	
Nipple-sparing mastectomy with inframammary fold incision	26 (56.52%)
Nipple-sparing mastectomy with periareolar incision	4 (8.70%)
Skin-reducing mastectomy with wise pattern incision	14 (30.43%)
Skin-sparing mastectomy	2 (4.35%)
**Occult cancer in risk-reducing mastectomy specimen**	
Yes	4 (8.7%)
No	42 (91.3%)
**Type of breast reconstruction (No,%)**	
Single-stage Prepectoral with ADM ^1^	16 (34.78%)
Single-stage Prepectoral without ADM ^1^	2 (4.35%)
Single-stage Dual-plane with ADM ^1^	6 (13.05%)
Single-stage Dual-plane with Bostwick’s Autoderm technique	4 (8.69%)
Two-stage Subpectoral (TE ^2^ followed by implant)	12 (26.08%)
Two-stage Dual-plane with ADM (TE ^2^ followed by implant)	2 (4.34%)
Other	4 (8.69%)
**TE ^2^ used (No, %)**	18 (39.13%)
**TE ^2^ size (mean ± SD, range) (cc)**	438.89 ± 108.33 (300–600)
**Implant used (No, %)**	44 (95.65%)
**Implant volume (mean ± SD, range) (cc)**	436.59 ± 81.42 (240–525)
**Implant shape (No, %)**	
Round	20 (43.48%)
Anatomical	24 (52.17%)
Unreported	2 (4.35%)
**Additional lipofilling (No, %)**	6 (13.04%)
**Lipofilling volume (mean ± SD, range) (cc)**	130 ± 44.34 (70–200)
**ADM ^1^ used (No, %)**	26 (56.52%)
Braxon^®^	16 (34.78%)
SurgiMend^®^	8 (17.39%)
Native^®^	2 (4.35%)
**Drain duration (mean ± SD, range) (days)**	8.83 ± 4.88 (4–20)
**Total drain amount ^3^ (mean ± SD, range) (mL)**	336.26 ± 287.25 (16–1139)
**Complications (No, %)**	10 (21.74%)
Major	8 (17.39%)
Minor	2 (4.35%)

^1^ Acellular dermal matrix ^2^ Tissue expander ^3^ Defined as the sum of daily collections from the first postoperative day until removal.

**Table 3:** Postoperative characteristics of the study population.

Variable	*n* = 16
Average postoperative pain ^1^ (mean ± SD, range) (NRS)	3.32 ± 2.13 (0–6)
Length of hospital stay (mean ± SD, range) (days)	9.38 ± 5.39 (4–24)
Patient-reported satisfaction (mean ± SD, range) (0 to 10 scale)	7.25 ± 1.28 (5–9)
Surgeon-reported outcome ^2^ (mean ± SD, range) (0 to 10 scale)	6.51 ± 1.82 (3.4–8.6)

^1^ Defined as the mean of patient-reported pain scores (NRS) in the first three postoperative days ^2^ Defined as the mean of surgeon-reported scores for each patient.

**Table 4:** Detailed surgical information about the forty-six operated breasts.

n	Side	Mastectomy (Incision)	Reconstruction	TE Size (cc)	BI Size (cc)	BI Shape	BI Manufacturer	Lipofilling Volume (cc)	ADM	Complications
1	R	NSM (IMF)	Two-stage submuscular	450	500	Round	Motiva	/	/	
2	R	NSM (IMF)	One-stage prepectoral	/	475	Round	Motiva	/	Braxon^®^	
3	L	NSM (IMF)	One-stage prepectoral	/	475	Round	Motiva	/	Braxon^®^	
4	R	NSM (IMF)	One-stage prepectoral	/	360	Anatomical	Polytech	/	/	
5	R	SRM	One-stage prepectoral	/	440	Anatomical	Mentor	/	Braxon^®^	
6	L	SRM	One-stage prepectoral	/	525	Round	Motiva	/	Braxon^®^	
7	R	NSM (IMF)	One-stage prepectoral	/	440	Anatomical	Mentor	/	Braxon^®^	
8	L	NSM (IMF)	One-stage prepectoral	/	440	Anatomical	Mentor	/	Braxon^®^	
9	R	SRM	One-stage prepectoral	/	475	Round	Motiva	/	Braxon^®^	Skin necrosis
10	L	SRM	One-stage prepectoral	/	475	Round	Motiva	/	Braxon^®^	Skin necrosis
11	R	NSM (P)	Two-stage submuscular	350	500	Round	Mentor	/	/	
12	R	NSM (IMF)	Two-stage submuscular	300				/	/	Baker grade III caspular contracture w/TE removal
13	R	NSM (P)	One-stage dual-plane	/	280	Anatomical	Allergan	/	Native^®^	
14	R	NSM (IMF)	Two-stage submuscular	500	520	Anatomical	Allergan	/	/	
15	R	NSM (T)	One-stage dual-plane	/	400	Anatomical	Allergan	/	SurgiMend^®^	
16	L	NSM (T)	One-stage dual-plane	/	400	Anatomical	Allergan	/	SurgiMend^®^	
17	R	SRM	Two-stage submuscular	400	520	Anatomical	Allergan	70	/	
18	L	SSM	Two-stage submuscular	400	520	Anatomical	Allergan	120	/	Partial NAC necrosis (minor complication)
19	R	NSM (IMF)	One-stage dual-plane	/	360	Anatomical	Allergan	/	/	
20	L	NSM (IMF)	One-stage dual-plane	/	360	Anatomical	Allergan	/	/	
21	R	NSM (IMF)	Two-stage dual-plane	350	310	Round	Allergan	/	SurgiMend^®^	
22	R	SRM	Other	600	475	Round	Motiva	200	SurgiMend^®^	Skin necrosis
23	L	SRM	Other	600	475	Round	Motiva	/	/	
24	R	NSM (IMF)	Two-stage submuscular	450	500	Round	Motiva	/	/	
25	R	NSM (IMF)	One-stage prepectoral	/	475	Round	Motiva	/	Braxon^®^	
26	L	NSM (IMF)	One-stage dual-plane	/	360	Anatomical	Allergan	/	/	
27	R	NSM (IMF)	Two-stage dual-plane	350	310	Round	Allergan	/	SurgiMend^®^	
28	R	NSM (IMF)	Two-stage submuscular	300				/	/	Baker grade IV caspular contracture w/TE removal
29	L	SRM	Other	600	475	Round	Motiva	/	/	
30	R	SRM	One-stage prepectoral	/	440	Anatomical	Mentor	/	Braxon^®^	
31	L	SRM	One-stage prepectoral	/	525	Round	Motiva	/	Braxon^®^	
32	R	NSM (IMF)	Two-stage submuscular	500	520	Anatomical	Allergan	/	/	
33	R	NSM (T)	One-stage dual-plane	/	400	Anatomical	Allergan	/	SurgiMend^®^	
34	L	NSM (T)	One-stage dual-plane	/	400	Anatomical	Allergan	/	SurgiMend^®^	
35	L	SRM	One-stage prepectoral	/	475	Round	Motiva	/	Braxon^®^	Skin necrosis
36	R	NSM (P)	Two-stage submuscular	350	500	Round	Mentor	/	/	Partial NAC necrosis (minor complication)
37	R	SRM	Other	600	475	Round	Motiva	170	SurgiMend^®^	Skin necrosis
38	R	NSM (P)	One-stage dual-plane	/	280	Anatomical	Allergan	/	Native^®^	
39	R	SRM	Two-stage submuscular	400	520	Anatomical	Allergan	90	/	
40	L	SSM	Two-stage submuscular	400	520	Anatomical	Allergan	130	/	
41	R	NSM (IMF)	One-stage dual-plane	/	360	Anatomical	Allergan	/	/	
42	R	NSM (IMF)	One-stage prepectoral	/	440	Anatomical	Mentor	/	Braxon^®^	
43	L	NSM (IMF)	One-stage prepectoral	/	440	Anatomical	Mentor	/	Braxon^®^	
44	R	SRM	One-stage prepectoral	/	475	Round	Motiva	/	Braxon^®^	Skin necrosis
45	L	NSM (IMF)	One-stage prepectoral	/	475	Round	Motiva	/	Braxon^®^	
46	R	NSM (IMF)	One-stage prepectoral	/	360	Anatomical	Polytech	/	/	

BI: breast implant; IMF: inframammary fold; P^:^ periareolar; T: inverted T; TE: tissue expander; ADM: acellular dermal matrix; NAC: nipple-areola complex.

**Table 5:** Comparison between single-stage and two-stage reconstruction.

Variable	Single-Stage (Prepectoral/Dual Plane Cohort)	Two-Stage (TE-Assisted Submuscular Cohort)	Mean Difference (95% CI)	*p*-Value
Age	47.9	52.0	−4.1 (−10.3–2.1)	0.1783
BMI	24.6	25.4	−0.8 (−5.9–4.4)	0.7455
Obesity	11.1%	28.6%	−17.5% (−64.9–30.0%)	0.4348
Smoking status	11.1%	14.3%	−3.2% (−4.2–3.6%)	0.8635
Alcohol consumption	0.0%	28.6%	−28.6% (−7.6–18.8%)	0.1820
Coffee consumption	66.7%	57.1%	9.5% (−47.3–66.4%)	0.7223
Diabetes mellitus	0.0%	0.0%	–	–
Previous chemotherapy	22.2%	42.9%	−20.6% (−75.4–34.1%)	0.4258
Previous radiotherapy	11.1%	85.7%	−74.6% (−113.7–−35.5%)	**0.0012**
Previous hormonal therapy	0.0%	42.9%	−42.9% (−90.6–4.9%)	0.0716
Previous breast cancer	33.3%	85.7%	−52.4% (−99.8–−5.0%)	**0.0329**
Previous breast surgery	33.3%	85.7%	−52.4% (−99.8–−5.0%)	**0.0329**
Previous SLNB	0.0%	71.4%	−71.4% (−118.8–−24.0%)	**0.0117**
Previous ALND	0.0%	14.3%	−14.3% (−48.1–19.5%)	0.3506
Previous ovarian cancer	11.1%	0.0%	11.1% (−14.5–36.7%)	0.3466
Previous prophylactic BSO	44.4%	14.3%	30.2% (−18.7–79.1%)	0.2057
Current breast cancer in contralateral breast	44.4%	71.4%	−27.0% (−81.8–27.8%)	0.3080
Contralateral therapeutic mastectomy	44.4%	71.4%	−27.0% (−81.8–27.8%)	0.3080
Additional lipofilling	0.0%	33.3%	−33.3% (−72.7–6.08%)	0.0856
ADM use	78.6%	22.2%	56.4% (17.0–95.7%)	**0.0078**
Drain duration	8.4	9.4	−1.0 (−5.1–3.1)	0.6141
Total drain amount	271.9	436.3	−164.4 ( −470.6–141.8)	0.2602
Complications	14.3%	33.3%	−19.1% (−60.5–22.4%)	0.3406
Postoperative pain	3.06	3.67	−0.6 (−2.1–0.90)	0.3944
Hospital stay	8.6	10.4	−1.9 (−7.6–3.9)	0.4923
Occult cancer in RRM	0.0%	22.2%	−22.2% (−55.5–11.0%)	0.1649
Implant volume in RRM	413.2	477.5	−64.3 (−133.6–5.1)	0.0671
Current SLNB in contralateral breast	22.2	71.4	−49.2% (−100.3–1.9%)	0.0578
Current ALND in contralateral breast	0.0%	14.3%	−14.3% (−48.1–19.5%)	0.3506
Patient-reported satisfaction	7.5	7.0	0.5 (−2.0–3.0)	0.6252
Surgeon-reported outcome	7.2	5.8	1.4 (−1.8–4.7)	0.3095

SLNB: sentinel lymph node biopsy; ALDN: axillary lymph node dissection; RRM: risk reducing mastectomy; TE: tissue expander; ADM: acellular dermal matrix; BSO: bilateral salpingo-oophorectomy. Bold numbers correspond to statistically significant values (*p* < 0.05).

**Table 6:** Summary and univariate analysis of postoperative complications.

	Total	Single-Stage (Prepectoral/Dual Plane Cohort)	Two-Stage (TE-Assisted Submuscular Cohort)	*p*-Value
No. of breasts	46	28	18	–
Overall complications (No,%)	10 (21.7)	4 (14.3)	6 (33.3)	0.3406
Seroma (No,%)	0 (0.0)	0 (0.0)	0 (0.0)	–
Hematoma (No,%)	0 (0.0)	0 (0.0)	0 (0.0)	–
Infection (No,%)	0 (0.0)	0 (0.0)	0 (0.0)	–
Wound dehiscence (No,%)	0 (0.0)	0 (0.0)	0 (0.0)	–
Capsular contracture (No,%)	2 (4.4) *	0 (0.0)	2 (11.1) *	0.3466
Major skin/NAC necrosis (No,%)	6 (13.0)	4 (14.3)	2 (11.1)	0.8320
Minor skin/NAC necrosis (No,%)	2 (4.4)	0 (0.0)	2 (11.1)	0.3466
Implant loss (No,%)	2 (4.4) *	0 (0.0)	2 (11.1) *	0.3466
Red breast syndrome (No,%)	0 (0.0)	0 (0.0)	0 (0.0)	–
Rippling/Wrinkling (No,%)	0 (0.0)	0 (0.0)	0 (0.0)	–
Implant malposition (displacement/rotation)	0 (0.0)	0 (0.0)	0 (0.0)	–

NAC: nipple-areola complex; TE: tissue expander * Capsular contracture leading to implant removal (both complications occurred in the same patient).

**Table 7:** Univariate analysis of risk factors for postoperative complications (OR, mean difference and *p*-values).

	OR (95% CI)	Mean Difference (95% CI)	*p*-Value
Staged reconstruction	2.85 (0.34–29.63)		0.3428
Age		0.51 (−4.20–5.22)	0.8194
BMI		1.22 (−3.19–5.64)	0.5403
Obesity	0.00 (0.00–6.54)		1.0000
Active smoking	9.66 (0.59–350.34)		0.1073
Alcohol consumption	0.00 (0.00–13.13)		1.0000
Coffee consumption	1.19 (0.14–12.11)		1.0000
Diabetes mellitus	–		–
Previous chemotherapy	1.31 (0.13–11.29)		1.0000
Previous radiotherapy	2.27 (0.27–23.27)		0.6175
Previous hormonal therapy	4.84 (0.38–63.52)		0.1937
Previous breast cancer	1.19 (0.14 –12.11)		1.0000
Previous breast surgery	1.19 (0.14 –12.11)		1.0000
Previous SLNB	4.81 (0.54–53.00)		0.1421
Previous ALND	3.91 (0.09–174.42)		0.3953
Previous ovarian cancer	0.00 (0.00–68.40)		1.0000
Previous prophylactic BSO	2.85 (0.34–29.63)		0.3428
Current breast cancer in contralateral breast	0.33 (0.01–3.23)		0.6106
Contralateral therapeutic mastectomy	0.33 (0.01–3.23)		0.6106
ADM use	1.19 (0.14–12.11)		1.0000
Drain duration		6.10 (−2.41–14.61)	0.1203
Total drain amount		148.14 (−247.50–543.79)	0.3810
Postoperative pain		0.42 (−0.84–1.68)	0.4728
Hospital stay		7.32 (−3.36–18.00)	0.1348
Occult cancer in RRM	3.91 (0.09 –174.42)		0.3953
Implant volume		60.70 (12.02–109.37)	**0.0174**
TE size		−8.33 (−407.24–390.57)	0.9388
Current SLNB in contralateral breast	0.51 (0.02–5.25)		1.0000
Current ALND in contralateral breast	0.00 (0.00–68.40)		1.000

SLNB: sentinel lymph node biopsy; ALDN: axillary lymph node dissection; RRM: risk reducing mastectomy; TE: tissue expander; ADM: acellular dermal matrix; BSO: bilateral salpingo-oophorectomy. Bold numbers correspond to statistically significant values (*p* < 0.05).

## Data Availability

Not applicable.
